# Enteric Fever.—II
*A Lecture delivered at the London School of Clinical Medicine.


**Published:** 1912-10-26

**Authors:** Guthrie Rankin

**Affiliations:** Physician to the "Dreadnought" and the Royal Waterloo Hospitals.


### ENTERIC FEVER.*—II.

By GUTHRIE RANKIN, M.D., F.R.C.P., Physician to the "Dreadnought" and the Royal Waterloo Hospitals.

The most serious complications of enteric fever are intestinal hæmorrhage and perforation. They may occur together, and are most to be feared during the third week. Peritonitis is a common cause of death, and may arise either from perforation through the floor of a Peyer's patch, or from extension through the peritoneal coat or from inflamed mesenteric glands. When it occurs, peritonitis is not always ushered in with severe symptoms. It may develop insidiously, and may even escape detection. This is explained by two considerations: (a) the blunted sensations of the patient; and (b) the limited area of the perforation, which allows the infecting agents to reach the peritoneum very slowly. In suspicious cases persistent hiccough is a symptom of important significance.

### Further Complications.

Appendicitis may complicate the illness, either as an independent affection or as the result of a typhoid ulceration of the appendical walls. Myocarditis is responsible for many fatalities. All varieties of pulmonary and pleuritic inflammation may interrupt convalescence. Repeated rigors sometimes occur during the period of defervescence and, despite the alarm they cause, usually pass off without the development of any explanatory cause; they are probably dependent upon a slight and passing toxæmia. Venous thrombosis is always a danger to be feared; it occurs most frequently in the femoral veins. Arteritis and phlebitis are also met with occasionally in the course of convalescence. Periostitis or necrosis, most commonly involving the ribs, tibiæ, or femurs, are well-recognised complications. They occur most commonly in young patients in whom the osseous tissues have not reached their full development. Parotitis, though infrequent, is of serious, and often of fatal, import. Orchitis occasionally happens and is usually unilateral. Ulceration of the larynx occurs in a small proportion of cases, and is always a formidable incident. A sequela of enteric fever which is too often overlooked is cholecystitis. Its importance as regards the subsequent occurrence of gall-stones or other gall-bladder conditions cannot be too strongly emphasised. Many nervous system disorders are traced back to an attack of enteric fever, but it is always difficult to be sure how far cause and effect are directly related.

As illustrative of some of the more important points connected with the foregoing account of enteric fever, Dr. Mitchell has been kind enough to tabulate a few details of the last ten cases recorded in our wards. From this table you will note how prevalent persistent headache is as a premonitory symptom; it occurred in eight cases of the series. It is further noteworthy that in half the number of cases rigors are reported as an early manifestation. One case is illustrative of the occasional misleading onset of the disease to which reference has been made, it having been ushered in with all the ordinary phenomena of an acute broncho-pneumonia. Unusual frequency of relapse is indicated by a record of this occurrence in three out of the ten cases. On the other hand, hæmorrhage, which is probably a much less frequent manifestation than the text-book accounts indicate, was met with in only one patient. The fact that diarrhœa is much less frequent than was once believed—it used to be described as a cardinal symptom—is evidenced by its occurrence as a pronounced factor in two only, and as an incidental symptom in three of the cases, the remaining 50 per cent. being the victims of obstinate constipation.

Widal's agglutination test was positive in eight cases and negative in one; in the remaining patient the record of the result has not been noted. The mortality in this short list was at the rate of 10 per cent.

The total duration of the illness varied between the extremes of 34 and 99 days.

The morbid changes associated with enteric fever are, as you know, primarily situated in the lymphoid tissues of the intestine. The intestinal mucous membrane is more or less involved in a catarrhal process, upon the severity and extent of which the amount of diarrhœa in great measure depends. Peyer's patches and the solitary follicles exhibit a series of changes which differ according to the period of hyperæmia, ulceration, necrosis, or cicatricial repair at which they are observed. The patches and follicles near the ilio-cæcal valve are those most seriously involved, and the process spreads upwards. The mesenteric glands are usually hyperæmic, swollen and hard, especially in the neighbourhood of the intestinal lesions. The spleen is always enlarged; the liver and kidneys are congested; the heart is frequently soft and flabby, and the lungs may be œdematous or congested at their bases. The bone marrow shows changes similar to those in the lymphoid tissues of the bowel. There is no time to do more than refer to the pathology of the disease, but it is of some importance to remember that an attack of enteric fever may occur and produce none of the characteristic local lesions. Cases have been reported in which, with an entire absence of the usual typical intestinal changes, the *Bacillus typhosus* has been obtained post-mortem in pure culture from an enlarged mesenteric gland. Osler says: "The wide existence of the typhoid bacilli has been repeatedly shown in cases which had the clinical features of the disease but without lesions in the small intestines. Typhoid fever is no more primarily intestinal than is smallpox primarily a cutaneous disease."

The prognosis should always be guarded, par-

* A Lecture delivered at the London School of Clinical Medicine.

ticularly in the early stages. There is, perhaps, no disease in which the ultimate issue so much depends upon the careful and judicious management of the patient, and there is none other in which death threatens from so many quarters. The older the patient the more unfavourable the outlook. The disease is always more serious in hot than in cool weather. Hyperpyrexia is a very unfavourable symptom. Fat subjects never stand enteric fever well. The mortality in women is greater than in men. An abundant diuresis throughout the illness is a good prognostic feature. In cases where there is copious and repeated hæmorrhage the ultimate issue is specially uncertain. The occurrence of perforation makes a fatal issue almost certain, unless surgical measures can be adopted effectively and at once. Heart failure is the most serious risk of all in uncomplicated cases. A small and irregular pulse is ominous, and should always give rise to anxiety if it reaches or exceeds 140. Patients whose constitutions are undermined by the abuse of alcohol are especially liable to succumb to the disease.

Aiming at the Diagnosis.

The diagnosis, in a well-marked case, is very simple, especially after the first week, but a mild attack may make a firm diagnosis difficult.

When enteric fever is prevalent in a neighbourhood the occurrence of persistent headache, anorexia, insomnia, general feebleness, and epistaxis should excite suspicion that the sufferer is smitten with the disease. If to these early manifestations there become added a remittent temperature, a soft dicrotic pulse, and a slight hectic flush over the malar prominences, strength is given to the original misgivings, and these are finally confirmed by the occurrence of tympanitis, tenderness and gurgling in the right iliac fossa, the characteristic rose-rash, and a positive Widal reaction. The agglutination test of Widal is not usually obtainable before the sixth or seventh day of the illness and may be much later. It is most conclusive when clumping occurs in half an hour in a dilution of 1 in 30.

A blood examination sometimes enables a definite diagnosis to be arrived at earlier than by any other method of investigation. From 5 to 10 c.c. of blood should be withdrawn from the median basilic vein by means of a fine syringe and forthwith incubated in nutrient boullion. In positive cases a growth is usually obtained in from 24 to 48 hours.

Wunderlich's proposition in connection with a doubtful diagnosis should always be recalled; he said that any fever which on the second day reaches 104° F. is not enteric fever, nor is it enteric if the fever does not approach 104° F. on the evening of the fourth day; on the other hand, enteric fever may be diagnosed if in a middle-aged person suffering from an acute febrile attack the evening temperature on the fifth day, or within the first week, is between 103° and 105°, and alternates with morning temperatures which are 1.4° to 1.7° lower, unless some other disorder can be discovered to explain the height of the fever.

Enteric fever may be mistaken for other disorders, especially the gastric type of influenza, typhus fever, acute miliary tuberculosis, appendicitis, ulcerative colitis, infective endocarditis, and para-typhoid fever.

The last named may be quite indistinguishable from true enteric fever; the differentiation lies wholly in the recognition of the infecting organism which, though closely allied to, is not the true Eberth's bacillus. A patient in the typhoid state should always be suspected of a para-typhoid infection when the Widal reaction is repeatedly negative.

Treatment.

There is no specific treatment for enteric fever. Careful nursing and a suitable dietary are the essentials of management. The patient should be put to bed at the earliest possible moment in a large well ventilated room and in a house beyond suspicion of sanitary fault. This is important in private practice, because, if the drainage system or the water supply of the house where the patient was first taken ill is suspect, his removal is imperative. Milk should be the mainstay of the dietary in the large proportion of cases. Three pints distributed over twenty-four hours and given in repeated small quantities serves all the requirements of most cases. The milk may be scalded, or peptonised, or mixed with barley-water according to indications. These are to be obtained from careful and repeated examinations of the stools; if masses of curd are found means must be taken to provide for the better digestion of the milk. The weak point in an all-milk dietary is that it falls somewhat short in carbohydrates; this may be provided for by adding one teaspoonful of maltine to the milk three or four times a day. If after a time the milk becomes distasteful it may be made more palatable by flavouring with tea, coffee, orange, or lemon. In those cases where it cannot be tolerated, no matter how disguised, recourse must be had to whey, mutton or chicken broth, and jellies prepared from veal, mutton, and fowl. Eggs may be given in the form of albumen water. Cold water or ice to quench thirst should be permitted freely; indeed, the patient ought to be encouraged to drink water to the extent of two or three pints in each twenty-four hours. Alcohol is not necessary until there is delirium, subsultus tendinum, and indications of cardiac failure.

It has recently become the custom with certain physicians to advocate a much more liberal dietary, and some go the length of recommending solid food, particularly meat, whenever a genuine desire is expressed for it by the patient, and when his physical condition is such as to interpose no reasonable objection. It is very likely that the milk-alone dietary is more Spartan than is absolutely necessary, but it answers extremely well in this hospital, and we must not forget that the experience of many men goes to prove that relapse appears to be directly due, in many instances, to the too early administration of solid food. In favourable cases, though solid food may be of doubtful advantage, the gradual addition of soluble and easily assimilable substances to the milk—such as custards, jellies, blancmanges, junket, bread jelly, potato-cream, pounded fish, tea, coffee, cocoa, etc.

—is, in our experience here, not only justified but desirable. Two conditions, in my own practice, guide me to these additions: A normal morning temperature and an expression on the part of the patient of a desire for more food.

The Danger of Pyrexia.

Pyrexia is one of the phenomena of enteric fever which demands careful watching. Its persistence is not without danger and it ought in every case to be controlled by the local application of cold in one form or another. A bath is the best means of adoption and, when available, should be had recourse to whenever the temperature reaches 102° or 103°. The patient ought to be completely immersed in water of 85° to 70° F. for from fifteen to twenty minutes. When taken from the bath he should be wrapped in a dry sheet and put back to bed under a light blanket. It is often wise to administer a small quantity of brandy and hot water and to apply hot bottles to the feet so as to promote the circulation and overcome any depression produced by the cold. The number of baths must be regulated by the patient's condition and the behaviour of his temperature. They may be repeated every six or eight hours if necessary. The only decided contra-indications to this bath plan of treatment are hæmorrhage or peritonitis. When, for any reason, the cold water is objected to, the bath may be made lukewarm—at first 90° to 80° F., and gradually cooled 10° or 12° by the addition of cold water. Failing the bath the patient may be packed over the chest and abdomen with tepid or cold water cloths; or he may be surrounded by an ice-cradle. Antipyretics are of no use in reducing temperature unless in extreme cases where the application of cold produces no lasting effect upon the pyrexia. Where they are thus indicated quinine, being germicidal to the typhoid bacillus, is probably the best to choose and should be given in two doses of ten grains at six and again at ten o'clock in the evening.

With a view to limiting the activity of the bacillus, many intestinal antiseptics have been recommended. A few of the more popular are chlorine water and quinine, perchloride of mercury with perchloride of iron, repeated small doses of calomel, carbolic acid, salol, salicylate of bismuth, beta-naphthol, essential oil of cinnamon, and so on. The weak point in all such forms of medication is that the bacilli do not multiply in the intestinal canal alone, but grow as well in the blood, spleen, mesenteric glands, and intestinal walls. In these wards salol is the antiseptic chosen in all cases except those associated with copious diarrhœa, when it is replaced by small doses of Dover's powder with salicylate of bismuth. If the urine is found to contain bacilli, urotropine in ten-grain doses may be combined with salol.

Abdominal distension, when excessive, is relieved by the administration of charcoal or by the local application of turpentine stupes, or by enemata of valerian or turpentine, or by the passage of a long rectal tube.

A moderate diarrhœa may be left alone, but if the evacuations exceed four or five daily they should be controlled by the B.P. pill of lead and opium, or by enemata of starch and laudanum. Constipation, on the other hand, must be combated by a simple enema given every alternate morning, with an occasional small dose of calomel at night followed by a morning saline draught. Hæmorrhage of moderate amount need not excite alarm, but when copious it must be controlled by opium in full doses, combined with chalk mixture, gallic acid, hamamelis, ergotine, or adrenalin chloride. The occurrence of hæmorrhage is an indication for the addition to each pint of milk of twenty grains of calcium chloride. In cases where the hæmorrhage is so copious as to induce collapse, the subcutaneous or intravenous injection of saline solution must never be omitted. The occurrence of perforation demands immediate surgical interference as the only hope of saving life.

Threatened cardiac failure is the indication for the free administration of alcohol, accompanied, if need be, by the hypodermic injection of strychnine, digitaline, ether, or caffein. When there is persistent insomnia, paraldehyde, chloralamide, bromural, or trional may be given at bedtime.

A serum prepared from the horse is now used in some cases, and is stated to influence favourably the course of the disease. The usual dose is 10 c.c. Chantemesse combines serum injections with cold baths and claims to have achieved encouraging results, especially in patients to whom the serum has been administered during the first week of the illness.

Bed-sores must be guarded against, and, in most cases, it is wise to put the patient on a water-bed early in the course of his illness. When convalescence is established, an ordinary mixed diet may be gradually resumed and recovery of strength will be promoted by suitable tonics and change of air.

Infection and Sanitation.

Prophylactic measures of sanitation, and not least among them compulsory notification, have done much to reduce the prevalence of enteric fever. The disease, according to Collie's statement, "does not spread any great distance when the ventilation is good, the cubic space abundant, and the general sanitary arrangements satisfactory." When enteric fever is prevalent in a neighbourhood, drinking water and milk should be boiled before use, and the skin should be entirely removed from raw fruit before it is eaten. Food supplies from suspected sources of contamination are to be avoided, and all varieties of mineral water should be forbidden until it is ascertained that the water used in their manufacture has been boiled or condensed and that the bottles and corks have been properly sterilised. Persons exposed to special risks of infection should be inoculated with anti-typhoid vaccine at the earliest opportunity.

It is abundantly proved that persons who have suffered from enteric fever may convey infection to others for many years after they themselves have recovered. When it happens that one or more cases otherwise unexplained, occur among those who are in close association with such a person, he should be suspected and if the typhoid bacillus is found in the fæces or urine, or if Widal's agglutination test is positive, he must be isolated. He is what is known as a typhoid-carrier and may unconsciously infect water, milk, or other articles of diet, even if he is not directly a source of danger. His treatment is a matter of great difficulty, but he must be kept under observation and every effort made by the use of vaccines, with intestinal and urinary antiseptics, to free him from the presence of the offending organisms. When by such measures this freedom is not obtained, the question of draining his gall-bladder and bile-ducts may be entertained.

### Preventive Precautions.

The following preventive precautions should be adopted in the conduct of every case:—

1. The mattresses and pillows, or such of them as are likely to become soiled with discharges, should be protected by rubber covers.
2. The bed and body linen should be changed daily.
3. All linen, etc., removed from the patient must be wrapped immediately in a sheet wrung out of carbolic solution, 1-20, or 2 per cent. lysol, allowed to soak for six hours, and then boiled.
4. Feeding utensils, after use, must be washed at once in *boiling* water.
5. The evacuations, both of bowel and bladder, should be received into a bed-pan containing half a pint of carbolic acid or lysol solution, and after they are passed a further and larger quantity of the antiseptic solution should be added.
6. When death occurs burial should follow without undue delay, the body being freely sprinkled with strong carbolic acid and coffined within a few hours of death. Where consent can be obtained, cremation should always be preferred to burial.
7. When recovery takes place the room occupied by the patient and all its contents must be subjected to the most thorough disinfection according to approved methods.
8. The nurses in attendance should wear overalls and rubber gloves when their duties demand contact with the patient. The hands should be repeatedly washed in an antiseptic solution, and it is a safe precaution to rinse out the mouth and throat from time to time with a similar fluid of suitable strength.
9. Every care must be taken that the patient, after convalescence, or those who have been in attendance upon him, do not unwittingly become, though apparently well, carriers of infection.

Tabulated Details of Ten Cases.

| Onset | Complications | Bowels | Widal | Relapse | Duration | Result |
|---|---|---|---|---|---|---|
| Case 1: Headache for 3 weeks before admission. Rigors | None | Constipation | Positive | One | 87 days | Recovery |
| Case 2: Headache for 4 weeks before admission. Backache | None | Early diarrhœa; late constipation | Positive | None | 47 days | Recovery |
| Case 3: Headache for 3 days before admission. Muscular pain, anorexia | Œdema of larynx, endocarditis, cardiac failure | Constipation | Positive | None | 37 days | Death |
| Case 4: Headache for 7 days before admission. Rigors, anorexia, lassitude | Hæmorrhage | Constipation alternating with diarrhœa | Positive | Three | 99 days | Recovery |
| Case 5: Headache for 10 days before admission. Rigors, lassitude | None | Copious diarrhœa | Negative | None | 80 days | Recovery |
| Case 6: Abdominal pains for 15 days before admission. Cough | Acute bronchopneumonia at onset | Diarrhœa | Not recorded | None | 81 days | Recovery |
| Case 7: Headache for 10 days before admission. Rigors, vomiting | None | Constipation | Positive | One | 34 days | Recovery |
| Case 8: Rigors, languor, and occasional vomiting for 10 days before admission | Two violent rigors. Thrombosis of left femoral vein | Constipation | Negative on admission; positive 4 days later | None | 56 days | Recovery |
| Case 9: Headache for 3 weeks before admission. Pains in joints | (Female patient) pyosalpinx suspected. Operation | Regular | Positive | None | 83 days | Recovery |
| Case 10: Headache for 12 days before admission. Vomiting | None | Constipation | Positive | None | 78 days | Recovery |

Compiled by Dr. Mitchell.

